# From biology to therapy: current standards and emerging strategies in pleural mesothelioma

**DOI:** 10.3389/fonc.2026.1778121

**Published:** 2026-03-26

**Authors:** J. H. L. T. van Genugten, C. J. de Gooijer, Paul Baas

**Affiliations:** 1 Department of Thoracic Oncology, Antoni van Leeuwenhoek Hospital/Netherlands cancer Institute (AVL-NKI) - Netherlands Cancer Institute/Antoni van Leeuwenhoek Hospital, Amsterdam, Netherlands; 2 Department of Pulmonology, Leiden University Medical Center, Leiden, Netherlands

**Keywords:** anti-angiogenic therapy, clinical trials, combination therapy, immune checkpoint blockade, immunotherapy, pleural mesothelioma

## Abstract

Pleural mesothelioma (PM) is an aggressive malignancy with limited therapeutic options and poor long-term prognosis. Platinum-based chemotherapy has been the standard-of-care for most patients for decades. While the decoding of the genomic alterations in PM has triggered renewed interest in mesothelioma biology and targeted therapies, these have yet to reach clinical practice. The recent introduction of dual immune checkpoint blockade (ICB) with nivolumab plus ipilimumab has marked the first major advance in PM treatment for decades, yet, many patients do not respond to these drugs and durable long-term responses remain rare. A range of alternative treatment modalities are currently under clinical investigation in PM, including antibody-drug conjugates, bi-specific antibodies, chimeric antigen receptor T-cells, cancer vaccines, and oncolytic viruses. Here we review recent progress in the understanding of PM biology and evaluate relevant phase I-III clinical trials aimed at improving treatment for this disease. We also highlight ongoing and future clinical trials with promising candidates for PM therapy.

## Introduction

Pleural mesothelioma (PM) is an aggressive cancer of the mesothelial lining of the thoracic cavity that is associated with the inhalation of asbestos fibres (1, 2). Asbestos was historically used as a construction material across the world, although its production and sale have been severely restricted in many countries since the 1980s (3, 4). Unfortunately, due to the long latency time between exposure and disease (5) and the continuing presence of asbestos in many older buildings (6) the disease remains an ongoing clinical challenge.

While there have been some improvements in PM management in recent decades, the prognosis remains poor and traditional standard-of-care treatments only provide limited survival benefits (7, 8). The recent introduction of first-line immunotherapy has marked the first significant advance in PM treatment for decades (9–12), and a range of promising new therapeutic modalities have recently entered phase I and II trials for this disease.

Here we will review our current understanding of PM development, biology, and its historical standard-of-care treatment options. We will also discuss emerging strategies to improve clinical outcomes and highlight ongoing and future trials with promising candidates for the treatment of PM.

## Scope and methods

This narrative review provides an overview of the biology and recent therapeutic advances for pleural mesothelioma. We highlight ongoing research and emerging therapeutic strategies.

We identified relevant clinical trials published between January 2020 and October 2025 through a core PubMed search query: ‘mesothelioma AND (clinical trial [pt] OR randomized clinical trial [pt]) AND (2020/01/01:2025/10/01 [dp])’. Rayyan.ai software was used to screen the abstracts for inclusion in this review (13). We also identified any articles prior to 2020 relevant to the contents of this review through targeted PubMed searches and tracing reference lists from included articles. The clinicaltrials.gov database was screened for ongoing and future clinical trials in pleural mesothelioma.

## The biology of pleural mesothelioma

### Asbestos and mesothelioma development

Pleural mesothelioma is strongly associated with inhalation of asbestos particles and was first reported to be a cause of lung disease in the late 19th century (14). This causal link was later confirmed in both epidemiology (15, 16) and animal studies (17–19) leading to a ban on asbestos mining and products in many countries by the 1990s (3, 4).

Several hypotheses have been proposed for the carcinogenic effects of asbestos fibres (20). One of the more influential theories states that frustrated phagocytosis of the fibers by macrophages (21, 22) leads to chronic inflammation of the pleura (23, 24), excessive release of reactive oxygen species (25) and ultimately malignant transformation. This theory is supported by the observation that the protein HMGB1 - which is associated with necrosis and inflammation - is released from asbestos-stimulated mesothelial cells and promotes malignant transformation (26, 27).

Alternatively, the persistent asbestos fibers may lead to genome instability and cancer development by physically disrupting chromosome structure leading to chromosome breaks and aneuploidy (28, 29). In addition, the fibers may cause DNA damage by inducing pyrimidine oxidation and DNA base alkylation leading to mutations and malignant transformation (30, 31).

Importantly, not all mesotheliomas are linked to asbestos exposure. Germline heterozygous loss of the tumor suppressor BAP1 may lead to an inherited predisposition for developing a variety of tumors including mesothelioma: BAP1 tumor predisposition syndrome (BAP1-TPS) (32). While individuals with BAP1-TPS are more prone to asbestos-related PM (33–35), a recent analysis of 84 individuals with a BAP1-TPS-related PM found that only one of these had a known history of asbestos exposure (36).

### The histopathology of pleural mesothelioma

The histology of PM tumors is classified into two major subtypes: epithelioid or sarcomatoid (non-epithelioid), with some tumors displaying a mixed biphasic type with proportions of both epithelioid and sarcomatoid features (37, 38). These subtypes have prognostic value, with epithelioid histology generally indicating a superior survival compared with non-epithelioid subtypes (39).

Immunologically, the non-epithelioid subtypes are associated with increased PD-L1 expression and CD8^+^ T-cell infiltration, while epithelioid tumors are characterized by peritumoral CD4^+^ T-cells and CD20^+^ B-cells (40, 41). These differences likely influence immunotherapy responses and will be discussed in more detail below.

The precise link between tumor histology and underlying genetics and gene expression patterns remains incompletely understood. Tumor mutation burden is broadly similar between the epithelioid and non-epithelioid subtypes, and there are no exclusive genomic alterations linked to any specific subtype (42–44). However, BAP1-loss appears to be enriched among epithelioid tumors (45), whereas MTAP-CDKN2A/B loss is enriched among non-epithelioid tumors (44).

## Systemic treatments: from chemotherapy to immunotherapy

Since the late 1990s, many single-agent chemotherapy drugs have been tested in PM. Unfortunately, these treatments rarely achieved response rates higher than 20%, and their benefits were typically short-lived. The first major shift in PM treatment occurred in 2003, with the landmark phase III trial by Vogelzang et al. (7) demonstrating an overall survival benefit for a combination of platinum-based chemotherapy with the antifolate drug pemetrexed. This regimen improved median overall survival (mOS) to 12.1 months compared with 9.3 months for patients treated with platinum-monotherapy, corresponding to a 23% reduced risk of death over time (HR: 0.77, log-rank p = 0.020) (**Table 1**).

**Table 1:** Results from randomized phase III trials in pleural mesothelioma.

Trial number	Trial name	Design	Treatment (number of patients)	Response rate	PFS (median, months)	OS (median, months)
NCT00005636	Vogelzang et al. (2003)	Multi-center, randomized, single-blind	First-line pemetrexed + cisplatin (n = 226) *vs*. cisplatin (n = 222)	41% *vs*. 17%	5.7 *vs*. 3.9 months [HR: 0.68, log-rank p = 0.001]	12.1 *vs*. 9.3 months [HR: 0.77, log-rank p = 0.020]
NCT00651456	MAPS	Multi-center, randomized, open-label	First-line pemetrexed + cisplatin + bevacizumab (anti-VEGF-A) (n = 223) *vs*. pemetrexed + cisplatin (n = 225)	–	9.2 *vs*. 7.3 months [HR: 0.61, p < 0.0001]	18.8 *vs*. 16.1 months [HR: 0.77, p = 0.0167]
NCT02709512	ATOMIC-meso	Multi-center, randomized, double-blind	First-line pemetrexed + cisplatin + pegargiminase (n = 125) *vs.* pemetrexed + cisplatin + placebo (n = 124)	13.8% *vs.* 13.5%	6.2 *vs.* 5.6 months [HR: 0.65, p = 0.02]	9.3 *vs.* 7.7 months [HR: 0.71, p = 0.02]
NCT02899299	CheckMate 743	Multi-center, randomized, open-label	First-line nivolumab (anti-PD-1) + ipilimumab (anti-CTLA-4) (n = 303) *vs*. pemetrexed + cisplatin/carboplatin (n = 302)	40% *vs*. 43%	6.8 *vs*. 7.2 months [HR: 1.00]	18.1 *vs*. 14.1 months [HR: 0.74, p = 0.0020]
NCT02784171	IND-227	Multi-center, randomized, open-label	First-line pembrolizumab (anti-PD-1) + pemetrexed + cisplatin/carboplatin (n = 222) *vs*. pemetrexed + cisplatin/carboplatin (n = 218)	62% *vs*. 38%	7.1 *vs*. 7.2 months [HR: 0.80, p = 0.0372]	17.3 *vs*. 16.1 months [HR: 0.79, p = 0.0324]
NCT02991482	PROMISE-meso	Multi-center, randomized, open-label	Second-line pembrolizumab (anti-PD-1) (n = 73) *vs*. vinorelbine/gemcitabine (n = 71)	22% *vs*. 6%	2.5 *vs*. 3.4 months [HR: 1.06, p = 0.76]	10.7 *vs*. 12.4 months [HR: 1.12, p = 0.59]
NCT03063450	CONFIRM	Multi-center, randomized, double-blind	Second- or later-line nivolumab (anti-PD-1) (n = 221) *vs*. placebo (n = 111)	11% *vs*. 1%	3.0 *vs*. 1.8 months [HR: 0.67, p = 0.0012]	10.2 *vs*. 6.9 months [HR: 0.69, p = 0.0090]
NCT01907100	LUME-meso	Multi-center, randomized, double-blind	First-line nintedanib (multi-TKI) + pemetrexed + cisplatin (n = 229) *vs*. placebo + pemetrexed + cisplatin (n = 229)	45% *vs*. 43%	6.8 *vs*. 7.0 months [HR: 1.01, p = 0.914]	14.4 *vs*. 16.1 months [HR: 1.12, p = 0.538]
NCT03762018	BEAT-meso	Multi-center, randomized, open-label	First-line atezolizumab (anti-PD-L1) + bevacizumab (anti-VEGF-A) + carboplatin + pemetrexed (n = 200) *vs*. atezolizumab (anti-PD-L1) + carboplatin + pemetrexed (n = 200)	55% *vs*. 49%	9.2 *vs*. 7.6 months [HR: 0.72, p = 0.0021]	20.5 *vs*. 18.1 months [HR: 0.84, stratified log-rank p = 0.14]

A subsequent study (NCT00651456) also demonstrated the additional benefit of combining a VEGF inhibitor (bevacizumab) with the platinum/antifolate regimen (46), increasing mOS to 18.8 months in selected patients (**Table 1**). Unfortunately, this treatment combination was never submitted for regulatory approval. For patients with the non-epithelioid histological subtype, the addition of the arginine-depleting drug ADI-PEG20 (pegargiminase) may further improve the efficacy of platinum/antifolate-based chemotherapy. In the recent ATOMIC-meso phase III trial (47) (NCT02709512), this combination significantly improved mOS from 7.7 months to 9.3 months (p = 0.02) (**Table 1**).

Immunotherapy with ICB drugs was successfully tested and approved for solid cancers such as advanced melanoma in the 2010s (48, 49), leading to the initiation of several phase II studies evaluating these drugs in PM (50–52). Results from these trials supported a head-to-head phase III comparison of ipilimumab (anti-CTLA-4) plus nivolumab (anti-PD-1) versus traditional cisplatin plus pemetrexed (9). This CheckMate 743 study (NCT02899299) became the second major milestone in PM treatment, demonstrating a mOS of 18.1 months for the ipilimumab plus nivolumab arm compared with 14.1 months for the chemotherapy arm, with a 26% reduction in the risk of death over time (HR: 0.74, p = 0.0020) (**Table 1**). The durability of this benefit has since been confirmed in the five-year survival data of CheckMate 743, showing a 14% versus 6% 5-year survival for all patients (53).

Notably, a subgroup analysis of the CheckMate 743 cohort showed a markedly improved outcome for patients with the non-epithelioid histological subtype (mOS increased from 8.8 months on chemotherapy, to 18.1 months on ICB), whereas patients with the epithelioid subtype only showed a modest 2-month survival gain with the hazard ratio crossing unity (9). Although the trial was not powered for statistical analysis within these subgroups, it is now widely accepted that the non-epithelioid subtype should preferentially be treated with dual-agent ICB, while both ICB and chemotherapy remain reasonable options for patients with the epithelioid subtype. Importantly, only a subset of approximately 40% of PM patients respond to dual ICB treatment.

Patients should be informed about expected treatment benefits, their duration, and potential side effects which can differ between dual ICB and chemotherapy treatment. In the CheckMate 743 trial any-grade serious treatment-related adverse events were reported in 21% of patients treated with nivolumab plus ipilimumab and 8% of patients treated with platinum-based chemotherapy (9). ICB-treatment related side-effects may include infusion related reactions such as fever and exanthema, and flare-ups of pre-existing immune conditions such as rheumatoid arthritis, hepatitis, and ulcerative colitis. Moreover, patients should be monitored for hyper and hypofunction of the thyroid gland, diabetes type I, nephritis, pneumonitis and other immune mediated diseases.

To improve these outcomes, various combination strategies have been evaluated in phase III trials. The IND-227 study (54) (NCT02784171) investigated adding pembrolizumab (anti-PD-1) to standard chemotherapy, resulting in a modest mOS improvement compared with chemotherapy alone (17.3 *vs*. 16.1 months) (**Table 1**). This study confirmed that the benefit of immunotherapy is mainly seen in the non-epithelioid subgroup (HR: 0.57, 95% CI: 0.36-0.89). Results for pembrolizumab-monotherapy in the second-line setting (NCT02991482) have remained disappointing (55), as it did not outperform the investigator’s choice of therapy (10.7 *vs*. 12.4 months) (**Table 1**).

The activity of second- or later-line nivolumab-monotherapy compared to best supportive care (BSC) was confirmed in the CONFIRM trial (56) (NCT03063450), which showed an improved response rate of 11% *vs*. <1% andan improved mOS of 10.2 vs. 6.9 months (**Table 1**). Further subgroup analysis suggested a major benefit for the non-epithelioid subgroup (11.2 *vs*. 6.2 months), although the trial was not powered for such subgroup analyses.

## Targeting angiogenesis to enhance treatment outcomes

The observation that the VEGF-inhibitor bevacizumab improves outcomes when combined with platinum/antifolate-based therapy (46) led to a greater interest in combining anti-angiogenic agents with chemo- or immunotherapies. These efforts have focused primarily on monoclonal antibodies (mAbs) and small-molecule (multi-)TKIs that block the VEGF-VEGF receptor axis.

Studies on single-agent angiogenesis inhibition in PM have only shown minimal efficacy, with responses to cediranib (57) (NCT00243074), vatalanib (58) (NCT00053885), sunitinib (59) (NCT00392444), and nintedanib (60) (NCT02568449) ranging between 0 and 9% (**Table 2**). These results emphasize the need for combining angiogenesis inhibitors with chemo- and/or immunotherapy to optimize their benefits.

**Table 2:** Results from phase II trials for anti-angiogenic drugs in pleural mesothelioma.

Trial number	Trial name	Design	Treatment (number of patients)	Response rate	PFS (median, months)	OS (median, months)
NCT00243074	SWOG S0509	Single-center, single-arm	Second-line cediranib (VEGF-inhibitor) (n = 47)	9%	2.6 months [95% CI: 1.7-3.7 months]	9.5 months [95% CI: 5.6-10.7 months]
NCT00053885	CALGB 30107	Multi-center, single-arm	First- or later-line vatalanib (VEGF-inhibitor) (n = 47)	6%	4.1 months [95% CI: 2.2-5.3 months]	10.0 months [95% CI: 6.3-14.4 months]
NCT00392444	Laurie et al. (2011)	Multi-center, single-arm	First- or later-line sunitinib (multi-TKI) (n = 35)	3%	2.7 months (in first-line); 2.8 months (in second- or later-line)	6.7 months (in first-line); 8.3 months (in second- or later-line)
NCT02568449	Wozniak et al. (2023)	Multi-center, single-arm	Second- or third-line nintedanib (multi-TKI) (n = 20)	0%	1.8 months [95% CI: 1.6-3.0 months]	4.2 months [95% CI: 2.5-8.7 months]
NCT01907100	LUME-meso	Multi-center, randomized, double-blind	First-line nintedanib (multi-TKI) + pemetrexed + cisplatin (n = 44) *vs*. placebo + pemetrexed + cisplatin (n = 43)	57% vs. 44%	9.4 *vs*. 5.7 months [HR: 0.56, p = 0.010]	18.3 *vs*. 14.2 months [HR: 0.77, p = 0.319]
NCT03560973	RAMES	Multi-center, randomized, double-blind	Second-line ramucirumab (anti-VEGFR2) + gemcitabine (n = 82) *vs*. placebo + gemcitabine (n = 83)	6% *vs*. 10%	6.4 *vs*. 3.3 months [HR: 0.79, un-stratified log-rank p = 0.082]	13.8 *vs*. 7.5 months [HR: 0.71, unstratified log-rank p = 0.028]
NCT03654833	MiST4	Multi-center, single-arm	Second- or later-line atezolizumab (anti-PD-L1) + bevacizumab (anti-VEGF-A) (n = 26)	4%	–	–
NCT04287829	PEMMELA cohort 1	Single-center, single-arm	Second- or third-line pembrolizumab (anti-PD-1) + lenvatinib (multi-TKI) (n = 38) after first-line chemotherapy	58%	5.6 months [95% CI: 4.5-8.5 months]	12.8 months [95% CI: 10.9-17.6 months]
NCT04287829	PEMMELA cohort 2	Single-center, single-arm	Second- or third-line pembrolizumab (anti-PD-1) + lenvatinib (multi-TKI) (n = 20) after first-line nivolumab plus ipilimumab	60%	6.9 months [95% CI: 5.5-NR months]	14.1 months [95% CI: 9.0-NR months]
NCT05918107	Cheng et al. (2025)	Multi-center, single-arm	First-line BNT327 (anti-PD-L1 x anti-VEGF-A) (n = 23)	43.5%	11.8 months	Not reached

After promising results for the addition of bevacizumab to platinum/antifolate-based therapy (46) (NCT00651456) (**Table 1**), several additional studies investigated the combination of anti-angiogenics with chemotherapy. Notably, the phase II part of the LUME-meso trial (61) (NCT01907100), combining the multi-TKI nintedanib with cisplatin and pemetrexed in the first-line setting, showed a promising improvement in response rate (57% *vs*. 44%) compared with cisplatin/pemetrexed alone (**Table 2**). However, the subsequent randomized phase III part of this trial (62) (NCT01907100) was unable to confirm any improvements in response rate (45% *vs*. 43%) or risk of death over time (HR: 1.12, p = 0.538) (**Table 1**).

In the second-line setting, the randomized phase II RAMES trial (63) (NCT03560973) showed that adding the VEGFR2-targeted mAb ramucirumab to gemcitabine improved mOS from 7.5 months to 13.8 months (**Table 2**). Although subgroups were small, the strongest effect for adding ramucirumab was seen in the non-epithelioid subgroup (HR: 0.37, 90% CI: 0.18-0.78).

The efficacy of combining anti-angiogenesis drugs with ICB treatment has been demonstrated in several solid cancers, most notably in renal cell carcinoma (64–66). In mesothelioma, the phase II MiST4 trial (67) (NCT03654833) combined bevacizumab with the anti-PD-L1 drug atezolizumab, however, while the study met its primary endpoint of a 50% 12-week disease control rate, the overall response rate was low at 4% (**Table 2**). Combining nintedanib with pembrolizumab has shown more promising results, with the phase Ib PEMBIP trial (68) (NCT02856425) reporting an overall response rate of 23% and a 47% disease control rate (DCR) at 6 months in 29 evaluable patients with advanced mesothelioma. Upcoming phase II clinical trials are scheduled to evaluate combinations of sintilimab (anti-PD-1) with bevacizumab plus pemetrexed plus cisplatin (NCT06543069), and sintilimab with the anti-angiogenic multi-TKI anlotinib (NCT05188859) plus pemetrexed plus cisplatin (**Supplementary Table 1**).

Out of all second-line treatments after ICB tested to date, the combination of pembrolizumab with the multi-TKI, VEGF-targeting inhibitor lenvatinib has shown the most promising response rates, with the PEMMELA 1 and 2 phase II trials (69, 70) (NCT04287829) showing a 58% response rate in patients that progressed after chemotherapy (69), and a 60% response rate in patients that progressed after with first-line nivolumab plus ipilimumab (70) (**Table 2**). Notably, a significant proportion of patients with PM who progressed after first-line nivolumab plus ipilimumab treatment still responded to second- or third-line pembrolizumab plus lenvatinib, highlighting the potential of this drug combination as a second-line option for patients progressing on nivolumab plus ipilimumab.

There has so far only been one phase III trial evaluating the benefits of combining anti-angiogenics with immunotherapy drugs. The BEAT-meso trial (71) (NCT03762018) combined the VEGF-inhibitor bevacizumab with atezolizumab plus carboplatin and pemetrexed, demonstrating a non-significant improvement in mOS from 18.1 months to 20.5 months (**Table 1**). Importantly, this study found a clear and significant improvement in both PFS (HR: 0.43, p < 0.001) and OS (HR: 0.50, p = 0.0022) for patients with a non-epithelioid subtype, while no significant benefits were found in the epithelioid subtype. These results highlight the importance of histological subtype stratification in treatment choice.

Bi-specific antibodies designed to target both angiogenesis and immune checkpoints simultaneously provide an attractive new avenue for targeting tumor angiogenesis with potentially reduced toxicity. The first phase II trial (72) (NCT05918107) with the anti-PD-L1 x anti-VEGF-A bi-specific antibody BNT327 has shown promising initial results, with a 43.5% response rate, mPFS of 11.8 months, and mOS not yet reached (**Table 2**). Multiple trials are ongoing with bi-specifics targeting angiogenesis, including BNT327 (NCT05918107) and ivonescimab (anti-PD-1 x anti-VEGF-A) (NCT06840834 and NCT06875076) (**Supplementary Table 1**). Moreover, the bi-specific antibody cadonilimab (anti-PD-1 x anti-CTLA-4) is explored in combination with the anti-angiogenesis drug bevacizumab (NCT05930665) (**Supplementary Table 1**). Alternative bi-specific antibody designs undergoing clinical trials in PM will be discussed below.

## Targeted therapies in pleural mesothelioma

Large-scale next-generation sequencing projects have revealed multiple recurrent genomic alterations in PM (42, 43). Loss of the tumor suppressors BAP1, NF2, CDKN2A, and TP53 are typical of PM tumors, while activating oncogene mutations are notably rare in this cancer type. This absence of activating oncogene alterations directly impacts therapeutic development, as targeting such alterations has been highly successful in other cancers including HER2 in breast cancer (73) and EGFR in lung cancer (74).

### NF2-targeted therapies

NF2 is a tumor suppressor that regulates activity of the Hippo signaling pathway (75). Targeting NF2-deficiency through its downstream effectors YAP/TAZ/TEAD is currently the most promising strategy for targeted therapy in PM. A recent phase I/II trial demonstrated that the YAP/TEAD inhibitor VT3989 yielded an objective response of 32% with a disease control rate of 86% in patients treated with a clinically optimized dose (76) (**Table 3**). These results provide strong support for further clinical development of YAP/TEAD inhibitors for the treatment of NF2-deficient pleural mesothelioma.

**Table 3:** Results from phase II trials with emerging therapeutics for pleural mesothelioma.

Trial number	Trial name	Design	Treatment type	Treatment (number of patients)	Response rate	PFS (median, months)	OS (median, months)
NCT04665206	Yap et al. (2025)	Multi-center, open-label	YAP/TEAD inhibitor	Second- or later-line VT3989 (YAP/TEAD inhibitor) (n = 91 pleural mesothelioma; n = 44 non-pleural mesothelioma)	17% (32% for clinically optimized dose)	**-**	**-**
NCT02610140	ARCS-M	Multi-center, randomized, open-label	Antibody-drug conjugate	Second-line anetumab ravtansine (anti-MSLN x DM4-payload) (n = 166) vs. vinorelbine (n = 82)	9.6% vs. 7.3%	4.3 vs. 4.5 months [HR: 1.22, log-rank p = 0.86]	9.5 vs. 11.6 months [HR: 1.07, log-rank p = 0.66]
NCT03126630	Mansfield et al. (2024)	Multi-center, randomized, open-label	Antibody-drug conjugate	Second- or later-line anetumab ravtansine (anti-MSLN x DM4-payload) + pembrolizumab (anti-PD-1) (n = 18) vs. pembrolizumab (anti-PD-1) (n = 17)	11% vs. 6%	12.2 vs. 3.9 months [HR: 0.55, p = 0.1952]	–
NCT04300244	NIPU	Multi-center, randomized, open-label	Anti-cancer vaccine	Second- or later-line UV1 (anti-telomerase) vaccine + ipilimumab (anti-CTLA-4) + nivolumab (anti-PD-1) (n = 59) vs. ipilimumab (anti-CTLA-4) + nivolumab (anti-PD-1) (n= 59)	31% vs. 16%	4.2 vs. 4.7 months [HR: 1.01]	15.4 vs. 11.1 months [HR: 0.73]
NCT01291420	Berneman et al. (2025)	Single-center, single-arm	Cell-based anti-cancer vaccine	Second- or later-line WT1-mRNA/DC vaccine (n = 10 mesothelioma patients)	0%	–	28.2 months [95% CI: 5.6-63.0 months]
NCT03610360	DENIM	Multi-center, randomized open-label	Cell-based anti-cancer vaccine	Second- or later-line MesoPher (DCs loaded with PM cell lysate) + best supportive care (n = 88) vs. best supportive care (n = 88)	6% vs. 5%	5.4 vs. 3.2 months [HR: 0.90, log-rank p = 0.60]	16.8 vs. 18.3 months [HR: 1.10, log-rank p = 0.62]
NTR7060	MESOPEC	Single-center, single-arm	Cell-based anti-cancer vaccine	First- or later-line MesoPher (DCs loaded with PM cell lysate) followed by CRS-HIPEC (n = 16)	–	12 months [IQR: 5–23 months]	–
NCT01721018	Danson et al. (2020)	Multi-center, single-arm	Oncolytic virus	First- or later-line intrapleural HSV1716 (replication-restricted herpes simplex virus) (n = 13)	0%	–	–
EudraCT: 2015-0051430-13	Ponce et al. (2023)	Multi-center, randomized, open-label	Oncolytic virus	Second- or later-line ONCOS-102 (engineered adenovirus) + pemetrexed + cisplatin/carboplatin (n = 20) vs. pemetrexed + cisplatin (n = 11)	21% vs. 46%	8.5 vs. 8.3 months [p > 0.05]	16.6 vs. 18.3 months [p > 0.05]

### BAP1-targeted therapies

The loss of BAP1 likely occurs early in PM development (77) and has been the focus of much preclinical and translational research (78). BAP1 is a Polycomb-related epigenetic enzyme that deubiquitinates histone H2A at position K119 (79, 80), thereby making the chromatin more accessible to the transcriptional machinery (81). This epigenetic role leads to a dependence of BAP1-deficient PM cells on EZH2 (82), which is the catalytic subunit of Polycomb-repressive complex 2 (PRC2). While several clinical trials have aimed to exploit this dependency with EZH2- and HDAC-inhibitors, these attempts have thus far not yielded improved outcomes for patients (83, 84). Recent preclinical work indicates that combining EZH2-inhibitors with ATM- or FGFR-inhibitors may improve outcomes (85, 86), but these drug combinations have yet to be confirmed in human studies. An alternative strategy for targeting BAP1-deficient PM relies on BAP1’s role in the DNA damage response. Early studies indicated that BAP1 interacts with the DNA damage response protein BRCA1 (87) and may be sensitive to PARP-inhibitors (88, 89), however, this strategy has also shown limited efficacy in human clinical trials (90–92).

### CDKN2A-targeted therapies

Loss of the cell-cycle regulator CDKN2A has been associated with a more aggressive clinical behavior of PM (122). This alteration potentially makes PM cells more sensitive to CDK4/6 inhibitors, as was demonstrated in preclinical PM models (93). In clinical trials the CDK4/6 inhibitor abemaciclib has shown some efficacy for CDKN2A (p16ink4A)-deficient PM, with a 12-week disease control rate of 54% and a response rate of 12% (94), warranting further investigation in randomized trials.

## Antigen-directed therapies in pleural mesothelioma

The efficacy of many systemic treatments is limited by off-target toxicities and limited tumor-specific uptake of the drugs. Multiple strategies have been developed to achieve a more optimal on-/off-target ratio for drug uptake. Several of these strategies, including antibody-drug conjugates (ADCs), bi-specific antibodies, and chimeric antigen-receptor (CAR) T-cells have recently entered phase I and II testing in patients with PM.

Efforts at tumor-specific drug targeting have so far focused primarily on the membrane protein mesothelin (MSLN), which is specifically expressed in PM (95). However, while this target is highly specific for PM, MSLN is known to be subject to protease-mediated shedding (96), potentially making MSLN a sub-optimal drug target. Current efforts focus on optimizing drug design to evade this shedding behavior (97), and evaluating alternative tumor-specific antigen targets including TROP2 and B7-H3.

### Antibody-drug conjugates

ADCs consist of a tumor-specific targeting antibody, linked to a cytotoxic payload (e.g. a tubulin inhibitor or DNA-damaging agent). These compounds are designed to deliver their payload specifically to tumor cells, where the ADC is internalized leading to degradation and release of the cytotoxic payload to the tumor cell (98). By selecting antibody targets that are highly specific to tumor cells, with limited expression elsewhere, these compounds aim to limit off-target toxicity while maximizing their concentration in the tumor microenvironment.

Anetumab ravtansine is an ADC that consists of an antibody targeting MSLN, linked to a payload containing a potent tubulin inhibitor (DM4). Upon internalization and release of the payload, the compound disrupts microtubule polymerization and induces cell cycle arrest, and apoptosis (99). A first-in-human phase I study (100) (NCT01439152) in 148 patients, including 47 mesothelioma patients, demonstrated that the compound is generally well tolerated and showed encouraging anti-tumor efficacy with 31% partial response and 44% stable disease. These findings led to the randomized phase II ARCS-M trial (101) (NCT02610140) comparing anetumab ravtansine with vinorelbine in 248 MSLN-positive mesothelioma patients. Unfortunately, this trial did not report any significantly improved response rates (9.6% vs. 7.3%) or OS (HR: 1.07, log-rank p = 0.66) for anetumab ravtansine compared with vinorelbine (**Table 3**).

In an attempt to improve the efficacy of anetumab ravtansine, it was combined with pembrolizumab in a phase II study (102) (NCT03126630). While this trial showed a non-significant trend towards improved PFS compared with pembrolizumab-monotherapy (HR: 0.55, p = 0.1952) (**Table 3**), the trial was closed early due to results from the ARCS-M trial, limiting the statistical power of this cohort.

LMB-100 presents an alternative ADC design, combining a MSLN-targeting antibody with an engineered Pseudomonas exotoxin A payload. The safety and tolerability of this compound have been confirmed in a phase I trial (103) (NCT02798536). However, the subsequent phase II trial of LMB-100 followed by pembrolizumab (NCT03644550) was terminated early by the principal investigators due to the FDA’s approval of nivolumab plus ipilimumab as first-line treatment for PM.

There are currently two phase II trials recruiting PM patients, including a study on the TROP2-directed ADC sacituzumab govitecan (NCT06477419) and the CD30-directed ADC brentuximab vedotin (NCT03007030) (**Supplementary Table 1**). These compounds both target tumor-specific membrane proteins and have already shown promising phase III results in other cancers including metastatic breast cancer (104) and T-cell lymphoma (105).

Overall, while ADCs provide an attractive platform for tumor-specific drug delivery, results from early trials on anetumab ravtansine and LMB-100 have not yet demonstrated durable clinical benefits in PM. Alternative antibody targets and combining these ADCs with other chemo- or immunotherapies may improve the efficacy of these drugs.

### Bi-specific antibodies

An alternative tumor-specific strategy that exploits tumor-specific protein expression is to use bi-specific antibodies designed to activate an anti-tumor response specifically in the tumor microenvironment.

ABBV-427 is a bi-specific antibody targeting MSLN and CD40, leading to T-cell priming and a more inflammatory tumor microenvironment (106). Activation of this CD40-mediated immune response specifically in the tumor microenvironment may limit the significant toxicity observed with CD40 agonists in previous trials (107). In a phase I dose-escalation trial (106) (NCT02955251) in 59 mesothelioma patients, ABBV-427 showed an acceptable safety and toxicity profile. However, only 9 out of 25 patients treated at the recommended phase II dose achieved stable disease, with no responses observed. Combining this compound with other chemo- or immunotherapy drugs may be required to optimize its clinical potential.

Among currently recruiting clinical trials, bi-specific antibodies are among the most actively investigated treatment classes, with multiple phase II and III clinical trials recruiting patients (**Supplementary Table 1**). Most notably, a worldwide phase III clinical trial is recruiting patients to test the anti-CTLA-4 x anti-PD-1 antibody volrustomig combined with platinum-pemetrexed and compared with investigator’s choice treatment (NCT06097728). Additional phase II trials will evaluate ivonescimab (anti-PD-1 x anti-VEGFA; NCT06875076, NCT06840834), cadonilimab (anti-CTLA-4 x anti-PD-1; NCT06416930), and BNT327/PM8002 (anti-PD-L1 x anti-VEGFA; NCT05918107). Moreover, a phase II trial combining the bi-specific antibody cadonilimab with the angiogenesis inhibitor bevacizumab (NCT05930665) is also recruiting patients (**Supplementary Table 1**).

### Chimeric antigen-receptor T-cells

CAR-T-cells form the third major class of antigen-directed therapies under investigation in mesothelioma, although results are so far limited to phase I trials. A MSLN-targeted CAR-T cell followed by pembrolizumab was tested in a first-in-human phase I study (108) (NCT02414269) that included 25 patients. This treatment proved safe and tolerable, with a promising mOS of 23.9 months, providing a strong rationale for combining these treatments in upcoming phase II trials.

The phase I FAPME trial (109) (NCT01722149) tested local delivery of fibroblast activation protein (FAP)-directed CAR-T cells in the pleural cavity of four PM patients. The treatment was feasible in three out of four patients tested, with a good safety profile. Unfortunately, due to the low number of patients in this study, efficacy could not be evaluated.

Several phase I trials are currently recruiting patients with PM for treatment with CAR-T cells (**Supplementary Table 1**). These include several MSLN-targeted CAR-T-cells: TNhYP218 (NCT06885697), UCMYM802 (NCT06256055), SynKIR-110 (NCT05568680), and the GPC3/Mesothelin-CAR-γδ cells (NCT06196294). An alternative target that is under evaluation for CAR-T-cell development in PM is B7-H3, with the MT027 CAR-T-cell undergoing phase I trials (NCT06726564) (**Supplementary Table 1**).

## Anti-cancer vaccinations

Therapeutic anti-cancer vaccinations are designed to induce an adaptive immune-response against the tumor by presenting tumor-specific (neo-)antigens via vaccination. These vaccinations may either be traditional peptide- or RNA-based formulations or may be delivered through cell-based platforms. While anti-cancer vaccines have only shown limited efficacy in solid cancers so far, efforts are ongoing to combine these with immunotherapies to improve their efficacy (110).

### Anti-cancer vaccines

UV1 is an anti-cancer vaccine that targets the telomerase enzyme, which is essential for maintaining telomere length and is overexpressed in 90-100% of all PM cases (111). The phase II NIPU-trial (112) (NCT04300244) assessed the benefits of combining the UV1-vaccine with ipilimumab and nivolumab in 118 PM patients. While the trial did not meet its primary endpoints, it found a non-significant improvement in response rate (31% vs. 16%) and mOS (15.4 vs. 11.1 months) (**Table 3**).

### Cell-based anti-cancer vaccines

An alternative approach for anti-cancer vaccine delivery is via dendritic cells loaded with tumor-specific antigens. These dendritic cells may either be loaded with patient-specific neoantigens, or with antigens derived from cancer cell lines to optimize their breadth of targeting (110).

Wilms’ tumor protein 1 (WT) is a transcription factor that may be specifically activated in cancer cells, against which an autologous mRNA-loaded dendritic cell vaccine was developed. In a phase I/II trial (113) (NCT01291420) including 10 mesothelioma patients, this vaccine reported a DCR of 70%, although no objective responses were detected. Based on a favorable safety profile and a promising mOS of 28.2 months (range: 5.6-63.0 months) (**Table 3**), this trial provides a basis for future randomized trials, including an upcoming phase I/II trial (NCT05765084) combining WT1/DC vaccination with ICB and chemotherapy (**Supplementary Table 1**).

The MesoPher vaccine was developed based on dendritic cells loaded with tumor lysate from five mesothelioma cell lines (114). This vaccine was tested both as a monotherapy in the DENIM-trial (115) (NCT03610360) and in combination with cytoreductive surgery followed by hyperthermic intraperitoneal chemotherapy (CRS-HIPEC) in the MESOPEC-trial (116) (NTR7060) (**Table 3**). While MesoPher monotherapy showed some improvements in DCR compared with best supportive care (57% vs. 40%; p = 0.020), no significantly improved OS was observed (HR: 1.10, log-rank p = 0.62). Combining the vaccine with CRS-HIPEC was shown to be feasible, with evidence for increased tumor immunogenicity and T-cell activation and a promising mPFS of 12 months. Based on these results, combining the MesoPher vaccine with CRS-HIPEC or other modalities such as immunotherapy may provide a basis for future follow-up trials. The ENSURE trial (NCT05304208) is currently recruiting patients to assess the feasibility of MesoPher vaccination after standard-of-care chemotherapy and followed by extrapleural pleurectomy/decortication (eP/D) surgery (**Supplementary Table 1**).

## Oncolytic viruses

Oncolytic viruses are an investigational treatment option that is based on viruses that specifically target, infect, and kill tumor cells. Local delivery of such viruses, for example via intrapleural catheter or intra-tumoral injection, may offer an innovative strategy to enhance the anti-tumor immune response in mesothelioma. Clinical trials on these viruses are currently in early stages, but they have already shown some efficacy in tumor killing and indirect immune activation.

HSV1716 is a replication-restricted herpes simplex virus that was evaluated for safety and tolerability in 13 patients with PM (117) (NCT01721018), demonstrating a favorable safety profile with limited treatment-related adverse events. The trial achieved some early efficacy, with stable disease in 6 out of 12 evaluable patients, although no objective responses were reported (**Table 3**). Analysis of pleural fluids from these patients showed an activated immune response in 8 out of 11 evaluable patients, characterized by cytokines such as IFNy, TNFa, and IL-2. Earlier pre-clinical work in mice has shown that HSV1716 may synergize with PD-1 blockade (118), providing a potential avenue for future clinical studies.

The attenuated vaccinia virus GL-ONC1, which was renamed to Olvi-vec, has been tested in two phase I studies in mesothelioma (NCT01443260; NCT01766739), demonstrating safety and tolerability for intrapleural injection of the virus (119, 120). Analysis of matched pre- and post-treatment biopsies from 4 patients showed increased CD8^+^ T-cell infiltration in 3 patients, and increased CD8/FoxP3 ratio in 2 patients, indicating a generally pro-inflammatory immune effect that warrants further investigations in combination with immunotherapy.

The combination of oncolytic viruses with chemo- or immunotherapies may be an attractive approach to boost their efficacy and has recently been tested in a phase I/II trial (121) (EudraCT: 2015-0051430-13) with the engineered adenovirus ONCOS-102, expressing granulocyte-macrophage colony-stimulating factor (GM-CSF). While combining this virus with chemotherapy showed evidence for improved immune infiltration in the tumor (e.g. CD4+ T-cells, CD8+ T-cells, M1:M2 macrophage polarization), the effects on mPFS (8.5 *vs*. 8.3 months; p > 0.05) and mOS (16.6 vs. 18.3 months; p > 0.05) were disappointing (**Table 3**).

There is a limited number of ongoing or upcoming trials evaluating oncolytic viruses, with only one study (NCT06031636), combining the oncolytic adenovirus H101 with anti-PD-1 drugs currently scheduled (**Supplementary Table 1**). This trial should provide the first evidence on the efficacy of combining oncolytic viruses with ICB treatment in mesothelioma.

## Conclusions

While the introduction of ICB treatment has significantly improved outcomes for patients with PM, especially for those with the non-epithelioid histological subtype, there remains a clear unmet need for improved therapies. Based on insights from large, randomized phase III trials, single-agent first-line immunotherapy should be discouraged as its efficacy is limited, while combining these ICB drugs with anti-angiogenic agents may provide improved survival benefits. Importantly, results from the PEMMELA trial suggest that combining pembrolizumab with lenvatinib may be an effective second-line treatment option for PM and demonstrate that this treatment still achieves responses even after first-line ICB treatment.

A surge in new therapeutic modalities has entered clinical evaluation in PM in recent years, with multiple phase I/II studies on targeted treatments, ADCs, bi-specific antibodies, CAR-T-cells, anti-cancer vaccines, and oncolytic viruses. While these compounds have generally shown acceptable safety profiles, their modest efficacy remains a bottle-neck for proceeding with phase III trials. In this context, the upcoming phase III trials for the YAP/TEAD-inhibitor VT3989 and for volrustomig plus pemetrexed plus carboplatin (NCT06097728) may be the most anticipated trials for improving PM care. For other candidates, improved drug design and/or rational treatment combinations will be required to realize their promise and effectiveness as new treatments for PM.

## Supplementary material

Supplementary Table 1 Overview of phase I, II, and III clinical trials recruiting patients with PM.
