# Supplementary material for: From biology to therapy: current standards and emerging strategies in pleural mesothelioma
Source: Front Oncol. 2026 Mar 26;16:1778121. doi: 10.3389/fonc.2026.1778121 (PMC13061707; doi:10.3389/fonc.2026.1778121)
Supplement: Supplementary Table 1 — Overview of phase I, II, and III clinical trials recruiting patients with PM. [file Table1.docx]

**Suppl. Table 1: Overview of phase I, II, and III clinical trials recruiting patients with PM.**

| **Trial number** | **Status** | **Region** | **Design** | **Study phase** | **Enrollment (estimated)** | **Treatment type** | **Treatment** |
| --- | --- | --- | --- | --- | --- | --- | --- |
| NCT06543069 | Recruiting | China | Single-centre, single-arm | II | 28 | Immune checkpoint blockade + angiogenesis inhibitor | First- or later-line sintilimab (anti-PD-1) + bevacizumab (anti-VEGF-A) + pemetrexed + cisplatin |
| NCT05188859 | Recruiting | China | Multi-centre, single-arm | II | 29 | Immune checkpoint blockade + multi-RTK inhibitor | First-line sintilimab (anti-PD-1) + anlotinib (multi-TKI) + pemetrexed + cisplatin |
| NCT06875076 | Recruiting | China | Multi-centre, single-arm | II | 25 | Bi-specific antibody | Second-line ivonescimab (anti-PD-1 x anti-VEGF-A) + pemetrexed/gemcitabine/vinorelbine |
| NCT06840834 | Recruiting | France | Multi-centre, single-arm | II | 38 | Bi-specific antibody | Second- or third-line ivonescimab (anti-PD-1 x anti-VEGF-A) |
| NCT05918107 | Recruiting | China | Single-centre, single-arm | II | 50 | Bi-specific antibody | First-line BNT327/PM8002 (anti-PD-L1 x anti-VEGF-A) + pemetrexed + cisplatin/carboplatin |
| NCT05930665 | Recruiting | China | Single-centre, single-arm | II | 38 | Bi-specific antibody + angiogenesis inhibitor | First-line cadonilimab (anti-PD-1 x anti-CTLA-4) + bevacizumab (anti-VEGF-A) + pemetrexed + carboplatin |
| NCT06477419 | Recruiting | United States | Multi-centre, single-arm | II | 33 | Antibody-drug conjugate | Second- or later-line sacituzumab govitecan (anti-TROP-2 x topoisomerase inhibitor payload) |
| NCT03007030 | Recruiting | United States | Single-centre, single-arm | II | 55 | Antibody-drug conjugate | Second- or later-line brentuximab vedotin (anti-CD30 x monomethyl auristatin E payload) |
| NCT06097728 | Recruiting | Worldwide | Multi-centre, randomised, open-label | III | 825 | Bi-specific antibody | First-line volrustomig (anti-PD-1 x anti-CTLA-4) + pemetrexed + carboplatin vs. investigator's choice |
| NCT06416930 | Not yet recruiting | China | Multi-centre, single-arm | II | 59 | Bi-specific antibody | Second- or third-line cadonilimab (anti-PD-1 x anti-CTLA-4) + gemcitabine/vinorelbine/pemetrexed |
| NCT06885697 | Recruiting | United States | Single-centre, single-arm | I | 100 | CAR-T cells | Second- or later-line TNhYP218 CAR-T cells (MSLN-targeted) |
| NCT06256055 | Recruiting | China | Single-centre, single-arm | I | 24 | CAR-T cells | First- or later-line UCMYM802 (MSLN-targeted) |
| NCT05568680 | Recruiting | United States | Multi-centre, single-arm | I | 42 | CAR-T cells | Second- or later-line SynKIR-110 (MSLN-targeted) |
| NCT06196294 | Recruiting | China | Single-centre, single-arm | I | 30 | CAR-γδT cells | First- or later-line GPC3/​Mesothelin-CAR-γδT cells (GPC3/MSLN-targeted) |
| NCT06726564 | Recruiting | China | Single-centre, single-arm | I | 18 | CAR-T cells | Second- or later-line MT027 (B7-H3-targeted) |
| NCT05765084 | Recruiting | Belgium | Multi-centre, single-arm | I/II | 15 | Anti-cancer vaccine | First-line WT1/DC vaccine + atezolizumab + pemetrexed + cisplatin/carboplatin |
| NCT05304208 | Recruiting | Netherlands | Single-centre, single-arm | I | 16 | Anti-cancer vaccine | Second- or later-line MesoPher (DCs loaded with PM cell lysate) followed by eP/D surgery |
| NCT06031636 | Recruiting | China | Single-centre, single-arm | - | 15 | Oncolytic virus | Second-or later-line H101 (oncolytic adenovirus) + PD-1 inhibitor |
